# Effects of an online organizational intervention targeting managers in nursing homes on caregivers’ low back pain and managers’ work engagement: a cluster randomized controlled trial

**DOI:** 10.1093/joccuh/uiag051

**Published:** 2026-09-04

**Authors:** Naomichi Tani, Takeshi Ebara

**Affiliations:** Department of Ergonomics, Institute of Industrial Ecological Sciences, University of Occupational and Environmental Health, Japan, Iseigaoka 1-1, Yahatanishi-ku, Kitakyushu, Fukuoka, Japan; Department of Ergonomics, Institute of Industrial Ecological Sciences, University of Occupational and Environmental Health, Japan, Iseigaoka 1-1, Yahatanishi-ku, Kitakyushu, Fukuoka, Japan

**Keywords:** organizations, work engagement, low back pain, digital technology, caregivers, participatory approach

## Abstract

**Objectives:**

To examine whether an online organizational intervention targeting managers in nursing homes could promote occupational health activities and reduce caregivers’ low back pain.

**Methods:**

This 6-month, prospective, cluster randomized controlled trial was conducted between January and July 2024. Nursing homes in Fukuoka Prefecture, Japan were randomly assigned to an online program-intervention group (*n* = 25) or an active control group (*n* = 25). The intervention combined an original tool that comprised practical tips for the prevention of low back pain with a series of meetings delivered via an online meeting system. Caregivers’ low back pain intensity was assessed as the primary outcome using a Numerical Rating Scale. Using the Japanese version of the Utrecht Work Engagement Scale, managers’ work engagement was evaluated as a secondary outcome. Linear mixed models were applied under the intention-to-treat and per-protocol frameworks.

**Results:**

A total of 48 managers and 449 caregivers working in the facilities were included in the final analysis. Over the 6-month study period, no significant differences were observed between the intervention and active control groups in caregivers’ low back pain intensity or managers’ work engagement. Participation in the intervention among managers was moderate (61.1%), and only 6 managers attended all the intervention sessions (adherence rate: 25%).

**Conclusions:**

Online organizational interventions targeting nursing homes did not significantly improve caregivers’ low back pain or work engagement. High job demands, suboptimal adherence, and constraints inherent in the video conference system potentially attenuated the intervention’s effects.

## Teaser

**Key points**
 

- **What is already known on this topic:** Individual-level interventions, such as exercise programs and the use of lifting devices, potentially contribute to reducing low back pain among caregivers; however, evidence regarding the effectiveness of organizational interventions that target managers in nursing homes remains limited.
- **What this study adds:** This cluster randomized controlled trial examined an online organizational intervention that targets managers in nursing homes to promote occupational health activities and reduce caregivers’ low back pain; however, no significant improvements were observed over the 6-month study period.
- **How this study might affect research, practice, or policy:** Our findings highlight the importance of considering the intervention duration, adherence, and outcome selection, and suggest the need to adopt multilevel participatory approaches involving both managers and caregivers in future occupational health interventions in nursing homes.

## 1. Introduction

Work-related musculoskeletal disorders (WMSDs), particularly low back pain (LBP), represent one of the most common occupational health issues in the health care and social welfare sectors. WMSDs affect millions of health care workers worldwide and cause absenteeism, reduced productivity, and substantial medical costs.^1^ The prevalence of LBP among health care workers ranges from 40% to 80%, depending on whether weekly, monthly, annual, or lifetime prevalence is assessed.^2–5^ These prevalence rates are considerably higher than the approximately 25% that is typically observed in other occupational groups.^4^ The high prevalence of LBP among health care workers is attributed to a complex interplay of factors that are specific to health care settings, including physical (eg, manual patient handling), organizational (eg, time pressure and insufficient staffing), psychosocial (eg, high workload and emotional demands), and individual factors (eg, age, sex, and educational level).^1^^,^^6^

In Japan, health care workers, particularly caregivers in nursing homes, experience work-related LBP at a rate approximately 2.5 times higher than the average across all industries, making it a major occupational health issue.^7^ Japanese nursing homes typically admit individuals who require a high level of care, including those with physical disabilities resulting from illness or injury. Consequently, caregivers supporting people with disabilities are exposed to high physical and psychosocial workloads during their daily caregiving activities.^1^^,^^8^ Various preventive measures such as stretching exercises and training in patient-lifting techniques have been implemented; however, these approaches have shown limited effectiveness in reducing LBP among caregivers.^9^ Exercise programs that are implemented by the caregivers themselves during caregiving duties frequently face substantial challenges related to adherence,^10^ which suggests that individual-level exercise interventions alone are impractical for addressing LBP in this population.

Therefore, the Ministry of Health, Labour and Welfare, Japan, has recently recommended the use of lifting devices in nursing homes.^7^^,^^11^ However, evidence indicates that although the introduction of lifting devices can reduce WMSDs, including LBP,^12^ the use of lifting devices alone is insufficient when organizational factors, such as excessive workload and time pressure, remain unaddressed.^6^^,^^13^^,^^14^ Only a limited number of nursing homes in Japan have been able to systematically manage the multiple risk factors associated with caregivers’ LBP.^14^ Recently promulgated behavioral change theories suggest that improving the effectiveness of interventions aimed at modifying individual behaviors and habits requires not only direct engagement with individuals but also the use of social and organizational resources, including interactions with supervisors, managers, and related organizations as well as organizational culture.^15^ These findings highlight the need for practical workplace-improvement processes that can systematically and comprehensively identify the multifaceted risk factors for LBP in nursing homes and facilitate effective work-environment improvements.

Therefore, we examined whether caregivers’ LBP in nursing homes could be indirectly addressed through interventions targeting the managers. Specifically, we hypothesized that a web-based organizational intervention targeting managers in nursing homes would facilitate occupational health activities within their facilities and ultimately reduce LBP in caregivers. To test this hypothesis, we developed an original tool that comprises a collection of practical tips for LBP prevention and provided it to managers in nursing homes. Concurrently, we implemented an online intervention program to guide managers in the effective use of this tool and to facilitate the implementation of workplace-improvement strategies to address the complex risks of LBP in nursing home settings. The program was delivered through an online meeting system and included educational components on LBP prevention, opportunities for peer exchange among nursing-home managers, and structured support to encourage the implementation of occupational health activities in the respective facilities.

## 2. Methods

### 2.1 Study design and participants

This 6-month, prospective, cluster randomized controlled trial was conducted between January and July 2024, and was designed to evaluate the effectiveness of an online organizational intervention program targeting managers in nursing homes. The intervention aimed to promote occupational health activities within nursing homes and consequently reduce LBP in caregivers. Therefore, caregivers were included in the outcome evaluation. Participants were recruited from among managers and staff working in nursing homes in Fukuoka Prefecture, Japan. The inclusion criteria were employees of nursing homes (regular, contract, or hourly employees), aged 18-64 years, with at least 3 months of employment. Caregivers who had received medical treatment for LBP at baseline were excluded. To minimize contamination and potential performance bias, participants were instructed not to disclose their assigned intervention conditions to individuals outside their facility. Managers were aware of their assigned intervention condition because of the nature of the intervention. However, caregivers were not informed of facility allocation and were therefore blinded to group assignment throughout the study period. The required sample size for managers was calculated based on an assumed effect size of 0.2, two-sided significance level of .05, and statistical power of 0.80. The minimum sample size required was estimated as 42 participants.

This study was approved by the Institutional Review Board of the University of Occupational and Environmental Health, Japan (ER23-020). Written informed consent was obtained from all participants before enrollment. The trial was conducted in accordance with the CONSORT extension for cluster randomized trials^16^ and registered in the UMIN Clinical Trials Registry (UMIN000051939).

### 2.2 Outcomes

This study was based on the theoretical assumption that an organizational intervention targeting managers would improve caregivers’ LBP through a series of organizational changes.^17^ Specifically, the intervention was designed to enhance managers’ knowledge of occupational health management and workplace improvement methods. Improved knowledge was expected to facilitate the implementation of workplace improvement activities, thereby strengthening occupational health management practices, improving the physical and psychosocial work environment, and ultimately reducing caregivers’ LBP.^18^^,^^19^ Accordingly, caregivers’ LBP intensity was selected as the primary outcome because it represents the ultimate health outcome of the organizational intervention. The theoretical logic model underlying the intervention is presented in Figure S1. However, the intervention could potentially increase managers’ workload, thereby hindering the implementation of workplace improvement activities.^17^ Therefore, managers’ work engagement was included as a secondary outcome to assess whether participation in the intervention had any unintended adverse effects on managers’ psychological and motivational states.

The primary outcome was the caregivers’ LBP intensity, which was assessed using a self-administered Numerical Rating Scale (NRS). LBP represents a major occupational health issue in Japan^7^ and serves as an appropriate indicator of occupational health conditions in nursing facilities. Caregivers were asked to rate the intensity of their LBP over the preceding 4 weeks on an 11-point scale, ranging from 0 (no pain) to 10 (severe pain). The primary outcome was the caregivers’ LBP intensity, which was assessed longitudinally over a 6-month study period.

The secondary outcome was managers’ work engagement, which was assessed using the Japanese ultrashort version of the Utrecht Work Engagement Scale (UWES-3).^20^ The UWES-3 evaluates 3 dimensions of work engagement—Vigor, Dedication, and Absorption—using a 7-point Likert scale (score range: 0 [never] to 6 [always]), with higher scores indicating greater work engagement. Managers completed the UWES-3 at 4 time points during the study period. This secondary outcome was included to evaluate whether participation in the organizational intervention adversely affected the managers’ psychological and motivational states, which could hinder the implementation of workplace improvement activities.

### 2.3 Procedures and intervention

A cluster randomized controlled trial was conducted to evaluate the effects of an online organizational program (OP) on caregivers’ LBP and managers’ work engagement in nursing homes. At baseline, the participating nursing homes were randomly assigned to either the OP intervention or active control group to avoid contamination between conditions within the same facility. Randomization was performed at the facility level. The random allocation sequence was derived from a computer-generated random number procedure. Simple randomization with a 1:1 allocation ratio was used. The principal investigator enrolled participating nursing homes, generated the allocation sequence, and assigned nursing homes to the intervention conditions. Using a self-administered questionnaire, managers completed the UWES-3 at 4 time points (baseline and at 2, 4, and 6 months), approximately 15 minutes before the end of the workday. Similarly, caregivers reported their LBP intensity using the NRS at the same 4 time points during the 6-month study period (Figure 1).

**Figure 1:**
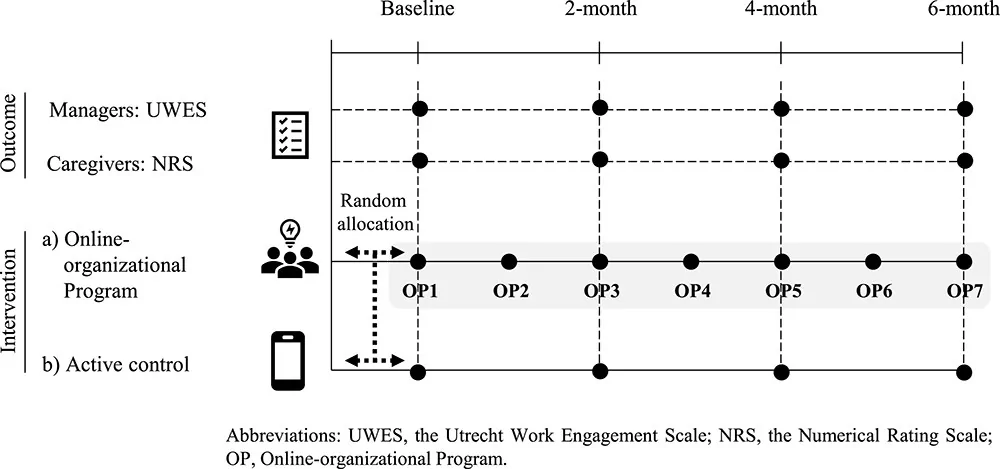
Study protocol and timeline of the intervention program. Managers and caregivers completed self-administered questionnaires (UWES-3 and NRS, respectively) at baseline and at 2, 4, and 6 months during the 6-month intervention period. NRS, numerical rating scale; UWES-3, 3-item Utrecht Work Engagement Scale.

The OP intervention adopted a participatory approach and was delivered via an online video meeting platform, with sessions lasting 30-60 minutes and conducted monthly throughout the study period. During the first session, managers completed a comprehensive educational program on workplace improvement that was delivered by certified ergonomists (Figure 1, OP1). In the second and third sessions, managers were paired to share information about their respective facilities and discuss occupational health challenges based on the knowledge gained during the initial educational session (Figure 1, OP2 and OP3).

In the fourth session, participants were introduced to the Organizational Diagnostic Tool (OrDiT)—an original toolkit that was designed to support workplace environment improvements—and were instructed on its use (Figure 1, OP4). OrDiT was developed based on ergonomic checkpoints that are conventionally used for participatory workplace improvements in health care and social welfare settings.^21^ Specifically, it is a tool that has been developed for nursing homes to enable managers to identify and prioritize areas for improvement across 5 domains: human resources, physical work environment, psychological stressors, staff education and training, and equipment and facilities, all of which are recognized risk factors for LBP.^1^^,^^14^ Furthermore, OrDiT provides guidance on directions for improvement, and lists relevant organizations and resources that can offer support or additional information. Nursing-home managers were emailed a PDF version of the OrDiT. Details of OrDiT are provided in the Supplementary Data (Appendix S1). For the next 2 months, managers implemented workplace improvements using OrDiT within their respective nursing homes. During the fifth and sixth sessions, the participants reported on the progress of these activities (Figure 1, OP5 and OP6). Prior to the final session, participants prepared and submitted written reports that summarized the activities they had undertaken. These reports included information on the workplace improvements implemented between OP4 and OP6, the OrDiT domains involved, the implementation process, challenges encountered, external resources used (if any), and photos of the activities (optional). The reported activities were categorized according to the corresponding OrDiT domains. In the seventh session, participants voted online to identify the nursing home that demonstrated the most effective workplace improvement activities (Figure 1, OP7). Adherence to the OP intervention was defined as the proportion of intervention sessions attended relative to the total number of sessions offered.

The active control group received general self-care information on the prevention of LBP via the official website of the Ministry of Health, Labour, and Welfare, Japan. The relevant website link was distributed via e-mail 4 times at 2-month intervals during the 6-month study period.

### 2.4 Statistical analysis

Descriptive statistics were used to summarize participants’ baseline characteristics. Linear mixed-effects models were used to examine the effects of OP intervention on managers’ work engagement and caregivers’ LBP intensity. The models included the intervention group (OP intervention vs active control), time (baseline and 2, 4, and 6 months), and their interactions as fixed effects. Nursing homes and individual participants were treated as random effects to account for the randomized cluster design and repeated measurements, respectively. The interaction between the intervention group and time constituted the primary indicator of the intervention effect. In case of significant between-group differences in baseline characteristics, those variables were included as covariates in the linear mixed-effects models.

Analyses were primarily conducted according to the intention-to-treat principle,^22^ whereby the participants’ data were analyzed according to their original group allocation regardless of their dropout status. Additionally, a per-protocol analysis was performed to examine the intervention effects among facilities in which managers attended 6 or more OP sessions and corresponded to an adherence rate of at least 80%. Missing data were handled using multiple imputation with predictive mean matching to reduce potential bias and preserve the statistical power.^23^ Five imputed datasets were generated. The imputation model included the outcome, intervention group, time point, age, sex, and years of work experience. The analyses assumed that data were missing at random. Parameter estimates and standard errors (SEs) were pooled across the 5 imputed datasets using Rubin’s rules, and 95% CIs were calculated from the pooled estimates and SEs. Participants without outcome data across all the assessment points were excluded from the analysis.

All analyses were conducted using R version 4.3.1 (R Foundation for Statistical Computing, Vienna, Austria).

## 3. Results

### 3.1 Participant flow and adherence

Details of the intervention program were distributed to 84 nursing homes, of which 50 agreed to participate in the study and were enrolled. The enrolled nursing homes were randomly assigned to either the OP intervention group (*n* = 25) or active control group (*n* = 25). Figure 2 illustrates the participant flow. Two managers who did not complete the questionnaire at any time were excluded from the analysis. Consequently, data from 48 participants were included in the final analysis. Informed consent was obtained from 692 caregivers working in the participating nursing homes. Of these, 401 and 291 caregivers belonged to facilities assigned to the active control and OP intervention groups, respectively. After excluding participants who were undergoing treatment for LBP, whose age or sex data were missing, had insufficient work experience, or were aged ≥65 years, we included data from 449 caregivers (270 and 179 in the active control and OP intervention groups, respectively) in the final analyses (Figure 2). All 7 planned online intervention sessions were delivered as scheduled. The overall participation rate of managers in the OP intervention was 61.1%. Ten managers participated in 6 or more intervention sessions (≥80% participation rate), whereas only 6 managers (25.0%) attended all 7 intervention sessions.

**Figure 2:**
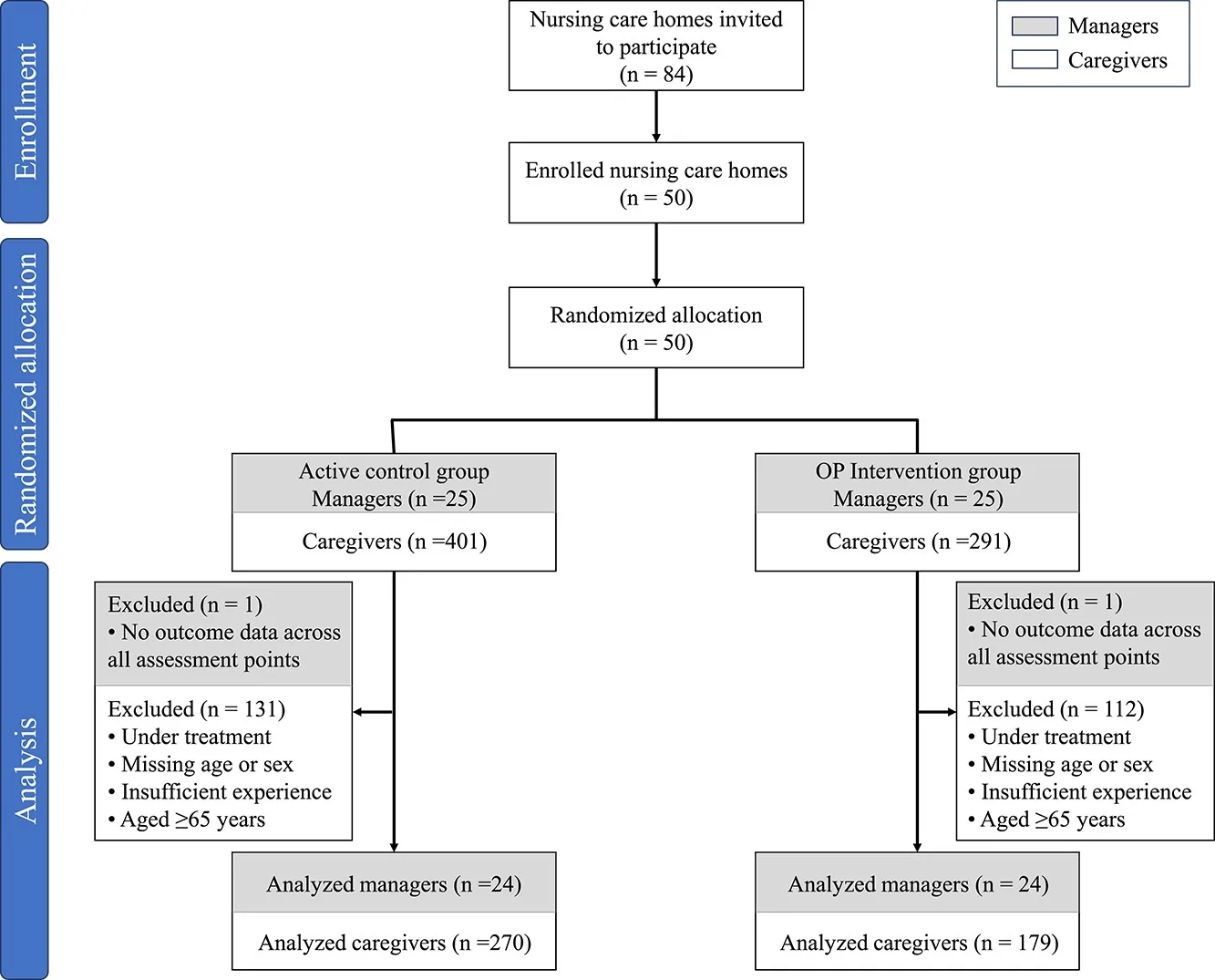
Flow diagram of managers and caregivers throughout the study period. OP, online-organizational program.

Between OP4 and OP6, 12 of the 24 intervention facilities (50.0%) reported implementing at least 1 workplace improvement activity. Among these 12 facilities, the reported activities addressed Human Resources in 5 facilities (41.7%), Physical Environment Resources in 2 (16.7%), Educational Resources in 4 (33.3%), and Support Resources in 2 (16.7%). No activities related to Stressor Management Resources were reported. One facility implemented activities across multiple OrDiT domains; therefore, the categories were not mutually exclusive. Overall, approximately half of the intervention facilities reported implementing at least 1 workplace improvement activity during the intervention period. Between OP6 and OP7, managers from the OP intervention group voted for the workplace improvement activities reported by these 12 facilities. Managers from 11 of the 24 intervention facilities (45.8%) participated in the voting.

The participant characteristics are presented in Table 1. Among managers, the mean age was 50.0 ± 10.8 years, 35.4% were female, and the mean length of work experience was 22.2 years. No significant differences in baseline characteristics were observed between the intervention and active control groups. Among caregivers, the mean age was 42.2 ± 11.6 years, 67.5% were female, and the mean length of work experience was 12.1 ± 7.7 years. Significant between-group differences were observed in age, sex, and work experience among the caregivers at baseline (Table 1). Therefore, these variables were included as covariates in a linear mixed-effects model.

**Table 1:** Baseline characteristics of managers and caregivers in nursing care homes.

Characteristics	Overall	Active control	OP intervention	*P* value
**Managers (*n* = 48)**				
**Age,**^**a**^ **y**	50.0 (10.8)	51.3 (11.6)	48.6 (10.0)	.397
**Sex: female**^**b**^	17 (35.4)	11 (45.8)	6 (25.0)	.227
**Years of experience**^**a**^	22.2 (9.1)	24.0 (10.6)	20.3 (7.1)	.162
**Caregivers (*n* = 449)**				
**Age,**^**a**^ **y**	42.2 (11.6)	41.3 (11.7)	43.7 (11.3)	.028
**Sex: female**^**b**^	303 (67.5)	166 (61.5)	137 (76.5)	<.001
**Years of experience**^**a**^	12.1 (7.7)	11.5 (7.7)	13.1 (7.5)	.025

Abbreviations: OP, online organizational program; SD, standard deviation.

a Mean (SD), *t-*test.

b *n* (%), chi-square test.

### 3.2 Intervention effects

The results of the linear mixed-effects model are presented in Table 2. No significant intervention effect on caregivers’ LBP intensity was observed over the 6-month study period in the intention-to-treat analysis (estimate = −0.08; 95% CI, −0.55 to 0.40; *P* = .753). The observed facility-level intraclass correlation coefficient (ICC) for the primary outcome was 0.064. Figure 3A illustrates the changes in the LBP intensity over time in both groups. Moreover, a per-protocol analysis of facilities with ≥80% intervention adherence showed no significant intervention effect on caregivers’ LBP intensity at 6 months (estimate = 0.13; 95% CI, −0.50 to 0.75; *P* = .693).

**Table 2:** Estimated intervention effects derived from linear mixed-effects models.

Outcome	Estimate ^a^	SE	95% CI	*P* value
**Caregivers’ low back pain intensity**	−0.08	0.24	−0.55 to 0.40	.753
**Managers’ work engagement (UWES-3)**	0.61	0.82	−1.03 to 2.25	.460

Abbreviations: CI, confidence interval; OP, online organizational program; SE, standard error; UWES-3, 3-item Utrecht Work Engagement Scale.

^a^Estimates represent between-group differences at 6 months calculated as the OP intervention group minus the active control group in the intention-to-treat analysis. Linear mixed-effects models included intervention group, time, and their interaction as fixed effects. Nursing care homes and participants were treated as random effects in the caregiver analysis, while managers were treated as random effects in the work engagement analysis.

**Figure 3:**
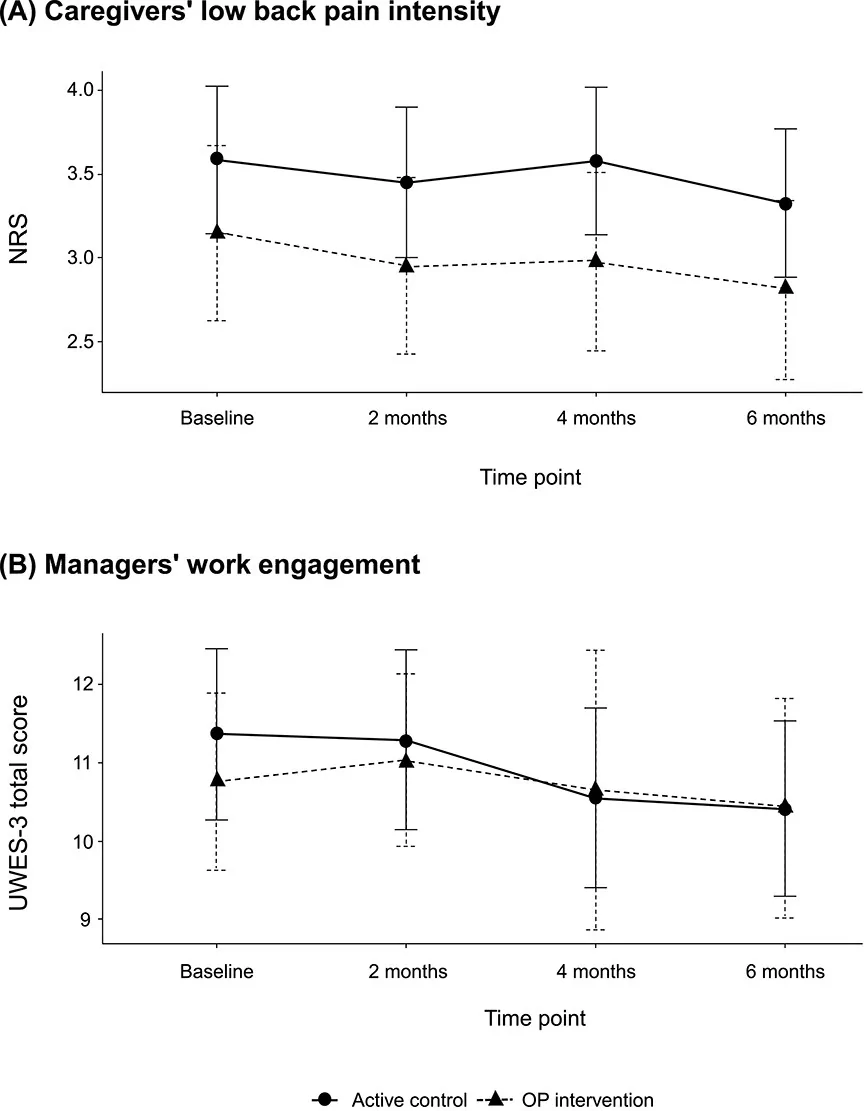
Model-based estimated marginal means and 95% CIs for (A) caregivers’ low back pain intensity and (B) managers’ work engagement during the 6-month study period. Estimated marginal means were derived from the linear mixed-effects models and pooled across 5 multiply imputed datasets. CI, confidence interval; NRS, numerical rating scale; UWES-3, 3-item Utrecht Work Engagement Scale.

Furthermore, no significant intervention effect on managers’ work engagement was observed during the 6-month study period (estimate = 0.61; 95% CI, −1.03 to 2.25; *P* = .460; Table 2). Changes in managers’ work engagement over time are presented in Figure 3B. Exploratory within-group comparisons showed slight reductions in caregivers’ LBP intensity and slight decreases in managers’ work engagement from baseline to 6 months in both the active control and OP intervention groups; however, none of these changes reached statistical significance after Bonferroni correction (Table S1).

## 4. Discussion

This study examined whether an online organizational workplace-improvement program targeting managers in nursing homes could improve LBP among caregivers working in these facilities. The online intervention program utilized in this study did not seem to have adverse effects on managers’ work engagement, as no significant differences were observed between the intervention and active control groups. Furthermore, contrary to our expectations, no significant differences in caregivers’ LBP intensity were observed between the intervention and active control groups during the 6-month study period.

One possible explanation for the limited effects observed in this study is that the high job demands faced by both nursing home managers and caregivers may have constrained the implementation of workplace-improvement activities. In Japan, nursing homes currently experience a chronic and severe shortage of personnel,^24^ and managers are frequently required to provide direct care services while simultaneously fulfilling their managerial responsibilities. Under such demanding working conditions, participation in additional organizational intervention activities may impose an additional burden on managers. Furthermore, synchronous online meeting systems require participants to be available at fixed times and locations,^25^ which potentially poses a substantial barrier for managers working in busy nursing-home settings. These constraints may partly explain the low adherence observed in this study, with only 25% of the managers attending all intervention sessions. Another possible explanation is that the online format of the intervention might have limited the development of strong interpersonal relationships and group dynamics among participating managers. Moreover, challenges that were commonly reported in previous studies on online meetings, such as managing distractions, multitasking, interruptions including background noise, and difficulties in fostering team cohesion,^26^ may also have been encountered during the online intervention sessions in this study. These factors might have attenuated the effectiveness of the intervention program. Further studies are needed to examine managers’ perceived workload and their engagement with online intervention programs to clarify whether these factors contributed to the limited intervention effects.

An alternative theory for the limited effects of the intervention on caregivers’ LBP may be the lack of direct engagement of the caregivers themselves in addition to the high job demands they face in nursing-home settings. Previous reviews of participatory workplace improvement and ergonomic interventions have emphasized the crucial role of workers’ active involvement in identifying risks, implementing improvements, and sustaining changes in the work environment.^27^^,^^28^ These approaches rely heavily on collective participation and group dynamics to achieve meaningful and long-lasting outcomes. From this perspective, interventions targeting managers alone, without directly involving frontline workers, may have been insufficient to activate the organization-wide participation and group processes that are indispensable to translate managerial initiatives into concrete behavioral and ergonomic changes among caregivers. This interpretation aligns with the core principles of participatory ergonomics, which posit that sustainable improvements in worker health are most likely to occur when both management and workers are actively involved in the intervention process.^29^^,^^30^ Therefore, future interventions in nursing-home settings may benefit from explicitly incorporating caregiver participation with managerial engagement.

A previous study suggested that the implementation of participatory workplace-improvement initiatives in nursing homes over a 3-year period was associated with improvements in caregivers’ LBP.^31^ The 6-month intervention period in the present study may have been insufficient for organizational interventions to translate into measurable effects among caregivers in nursing-home settings. Furthermore, although approximately half of the intervention facilities implemented at least 1 workplace improvement activity, these activities were introduced primarily during the final 3 months of the intervention. This relatively short implementation period may have been insufficient for organizational changes to become embedded in routine practice and subsequently influence caregivers’ LBP. Moreover, many activities focused on organizational systems and educational initiatives rather than direct modifications to the work environment, suggesting that the organizational changes may not have been sufficient to meaningfully influence the physical and psychosocial work environment within the 3-month implementation period.

Although changes in LBP intensity were used as an outcome measure in this study, no significant improvement in LBP intensity was observed, even in the previous 12-month participatory workplace-improvement intervention.^32^ Driessen et al^32^ reported that risk factors for LBP onset differed from those associated with the development of chronic LBP. Therefore, for workplace-improvement initiatives aimed at reducing occupational risk factors for LBP, the incidence of LBP (ie, the occurrence of new cases) may be a more appropriate outcome measure than changes in pain prevalence or intensity. Nonetheless, the active control condition in this study was not a no-treatment control but rather comprised self-care information for LBP prevention that was recommended by the Ministry of Health, Labour and Welfare, Japan. Therefore, the nonsignificant between-group differences suggest that the present study did not provide evidence that the OP intervention conferred additional benefits over the active control condition during the 6-month study period.

This study has some limitations that should be considered when interpreting the findings. First, the sample size calculation did not explicitly account for the cluster randomized design, which may have limited the statistical power. The observed facility-level ICC for the primary outcome was 0.064, indicating a modest degree of within-facility clustering and suggesting that the effective sample size was smaller than the nominal sample size. Second, the 6-month intervention period may have been insufficient for managerial workplace improvement activities to translate into measurable effects on caregivers’ LBP. Third, LBP intensity and work engagement were assessed using self-administered questionnaires. Finally, this study was conducted only in Japanese nursing homes, which may limit the generalizability of the findings to other settings.

## 5. Conclusions

In this cluster randomized controlled trial to evaluate an online organizational workplace-improvement program targeting managers in nursing homes to reduce caregivers’ pain (LBP), the intervention neither significantly improved caregivers’ LBP intensity over the 6-month study period nor adversely affected managers’ work engagement.

Therefore, although online managerial interventions are feasible in nursing-home settings, their effectiveness may be constrained by the high job demands, suboptimal adherence, and insufficient participatory engagement of frontline workers. Future interventions should consider multilevel approaches that combine managerial and caregiver participation, incorporate optimally designed implementation support, allow for longer follow-up periods, and carefully select appropriate health-related outcome measures to adequately evaluate intervention effects.

## Supplementary Material

Supplementary_Materials_uiag051

## Data Availability

Data used in this study will be shared by the corresponding author upon request.
