# Supplementary material for: Effects of an online organizational intervention targeting managers in nursing homes on caregivers’ low back pain and managers’ work engagement: a cluster randomized controlled trial
Source: J Occup Health. 2026 Sep 4;68(1):uiag051. doi: 10.1093/joccuh/uiag051 (PMC13674606; doi:10.1093/joccuh/uiag051)
Supplement: Supplementary file 1 [file supplementary_materials_uiag051.zip › Supplementary Appendix.docx]

**Supplementary Appendix**

**Summary of Overview of the Organizational Diagnosis Tool (OrDiT)**

### ***Purpose of OrDiT***

The Organizational Diagnosis Tool (OrDiT) was developed to support workplace improvement activities for the prevention of low back pain (LBP) among caregivers working in nursing care homes. The tool assists managers in identifying organizational factors associated with occupational health and selecting priority areas for workplace improvement.

### ***Structure of OrDiT***

OrDiT consists of 20 checklist items organized into five domains:

1. **Human Resources (2 items)**
   - Occupational health and safety management systems
   - Staffing arrangements and work schedules for safe care delivery
2. **Physical Environment Resources (4 items)**
   - Adequate working space around beds, toilets, and bathrooms
   - Obstacle-free walkways and circulation routes
   - Sufficient space for meal delivery and feeding assistance
   - Dedicated spaces for laundry management
3. **Stressor Management Resources (4 items)**
   - Mental health promotion activities
   - Psychological safety initiatives
   - Consensus-based care organization
   - Flexible scheduling arrangements
4. **Educational Resources (3 items)**
   - Education on LBP prevention and patient handling
   - Opportunities to practice using assistive devices
   - Providing opportunities and funding for professional development
5. **Support Resources (7 items)**
   - Supporting hygienic waste handling
   - Organized linen storage systems
   - Digitalization of care documentation
   - Electrically adjustable beds
   - Wheelchairs with removable armrests
   - Providing adequate lifting devices and sliding sheets
   - Assistive tools for reducing physical workload

### ***Assessment Procedure***

Managers evaluated each item using the following categories:

- ○ = Adequately implemented
- △ = Requires improvement
- – = Not applicable

Items identified as requiring improvement were prioritized and selected for workplace improvement activities.

### ***Workplace Improvement Process***

Managers were encouraged to:

1. Identify priority improvement areas using OrDiT.
2. Establish an internal implementation structure.
3. Assess required resources, budget, and personnel.
4. Utilize external support services when necessary.
5. Develop an implementation schedule.
6. Conduct workplace improvement activities.
7. Document and evaluate outcomes.

### ***Supporting Information for Workplace Improvement***

For each checklist item, explanatory information describing the rationale for workplace improvement was provided, together with practical tips for improvement activities and information on locally available services and resources.

The full Japanese version of OrDiT was used during the intervention and is available from the corresponding author upon reasonable request.
