# Supplementary material for: Effects of an online organizational intervention targeting managers in nursing homes on caregivers’ low back pain and managers’ work engagement: a cluster randomized controlled trial
Source: J Occup Health. 2026 Sep 4;68(1):uiag051. doi: 10.1093/joccuh/uiag051 (PMC13674606; doi:10.1093/joccuh/uiag051)
Supplement: Supplementary file 1 [file supplementary_materials_uiag051.zip › TableS1.docx]

| **Supplementary Table 1.** Exploratory within-group comparisons from baseline to 6 months for caregivers’ low back pain intensity and managers’ work engagement. | | | |
| --- | --- | --- | --- |
|  | Estimate | SE | p value |
| NRS |  |  |  |
| Active control | 0.29 | 0.15 | .317 |
| OP group | 0.37 | 0.18 | .247 |
| UWES-3 |  |  |  |
| Active control | 0.96 | 0.51 | .385 |
| OP group | 0.88 | 0.51 | .545 |
| Abbreviations: OP, online organizational program; SE, standard error; NRS, Numerical Rating Scale; UWES-3, 3-item Utrecht Work Engagement Scale. For low back pain intensity, positive estimates indicate reductions in pain severity. For work engagement, positive estimates indicate decreases in UWES-3 scores from baseline to 6 months. | | | |
